# Uniportal Laser-Assisted Video-Assisted Thoracoscopy (U-LA-VATS) for Lung Metastasectomy: Technical Description, Peri-Operative Results and Pertinent Literature Review

**DOI:** 10.3390/jcm13185346

**Published:** 2024-09-10

**Authors:** Carolina Sassorossi, Marco Chiappetta, Dania Nachira, Annalisa Campanella, Gloria Santoro, Giuseppe Calabrese, Chiara Scognamiglio, Antonio Giulio Napolitano, Alessia Senatore, Leonardo Petracca Ciavarella, Maria Letizia Vita, Stefano Margaritora, Filippo Lococo

**Affiliations:** 1 Thoracic Surgery, A, Gemelli University Hospital Foundation IRCCS, 00168 Rome, Italy; marco.chiappetta@policlinicogemelli.it (M.C.); dania.nachira@policlinicogemelli.it (D.N.); annalisa.campanella@guest.policlinicogemelli.it (A.C.); giuseppe93calabrese@virgilio.it (G.C.); kiasco1998@gmail.com (C.S.); antoniogiulionapolitano@gmail.com (A.G.N.); alessiasenatore2@gmail.com (A.S.); leonardo.petraccaciavarella@policlinicogemelli.it (L.P.C.); marialetizia.vita@policlinicogemelli.it (M.L.V.); stefano.margaritora@policlinicogemelli.it (S.M.); 2 Thoracic Surgery, Catholic University of Sacred Heart, 10123 Rome, Italy; 3 UOC di Chirurgia Generale, Fondazione Policlinico Universitario A. Gemelli IRCCS, 00168, Rome, Italy; gloria.santoro@policlinicogemelli.it

**Keywords:** lung cancer, laser-assisted surgery, uniportal VATS, surgery

## Abstract

Pulmonary metastasectomy (PM) is a well-established treatment that is able to contribute to the cure of oligometastatic cancer. Surgery should adopt the most lung-sparing approach possible to preserve pulmonary function (and, consequently, the quality of life) and to spare the lung for potential additional lung resections. In this framework, laser technology has been introduced in recent decades, but only few experiences combining laser technology with VATS approaches have been reported till now. The main focus of this manuscript is to report our institutional experience in performing lung-sparing laser-assisted PM by uniportal VATS (uniportal laser-assisted VATS: U-LA-VATS). The surgical technique and peri-operative results from our series of patients were herein presented and compared with the pertinent literature. **Methods**: Between March 2021 and November 2023, among 98 patients who underwent PM, a total of 24 patients (18 men (75%); 6 women (25%); mean age 61.4 years; age range 13–83 years) were treated with laser-assisted PM at our institution. Patients who underwent anatomical resection were excluded for the purpose of the analysis. The U-LA-VATS procedure adopted a modified laser-assisted lung resection technique for performing PM via VATS. Dedicated instruments are used, characterized by a long shape and a curved shape, with distal and proximal articulations. A surgical laser system (Thulium + Diodo OUTPUT 30–10 W, Quanta System S.p.a., Solbiate Olona, Italy) was used, and a 550-μm sterile optical fibre conducted through a specific thoracoscopic handpiece was introduced in the lowest part of the incision. Peri-operative results were analysed in all cohort and compared according to the surgical technique. Moreover, these results were compared with those reported in the literature. Comprehensive research of the literature was conducted on PubMed from 2000 to 2024. A review was performed and reported in line with the Preferred Reporting Items for Systematic Reviews and Meta-Analyses (PRISMA) statement. **Results**: In 12 cases (50%), thulium laser-assisted resection was performed using uniportal video-assisted thoracic surgery (VATS), and in the other cases (12, 50%), a (mini)thoracotomy access was adopted. In the thoracotomy group, the mean duration of surgery was 95 ± 57.7 min; meanwhile, it was 73.5 ± 35.5 in the uniportal VATS group. At the univariate analysis, this difference resulted to be statistically significant (*p* value 0.025). We did not observe intra-operative complications or remarkable malfunction of the laser system. We also did not report major complications after surgery; also the air-leak rate was 8.3% and 0% after thoracotomic and VATS procedures, respectively. Surgical margins were free from disease in all cases. Major and minor post-op complication rates were similar in both groups. The mean hospitalization after surgery was 2.9 ± 0.3 days for the uniportal VATS group and 3.7 ± 0.9 days for the thoracotomy group, this difference being statistically significant at the univariate analysis (*p* value = 0.015). **Conclusions**: U-LA-VATS is a safe and effective procedure, able to combine a parenchymal sparing exeresis with a mini-invasive approach. This procedure is associated with a shorter hospital stay compared with PM performed by a thoracotomic approach. Compared with the selected works for the review, our series is the only one describing the use of laser resection combined with a uniportal VATS approach.

## 1. Introduction

Pulmonary metastasectomy (PM) is a well-established treatment that is able to contribute to the cure of oligometastatic cancer [1]. The indication of PM is considered from both physiological and oncological points of view and on the basis of the eligibility of the patient to undergo surgery [2]. Kondo and coworkers, in 2005, postulated a few major criteria to take into account when planning a metastasectomy, which are still valid and considered nowadays: good risk profile for surgical intervention, primary site controlled, no other extrapulmonary metastasis or, if present, can be controlled by surgery or another treatment modality. Furthermore, surgery should be planned only when it is considered technically feasible to remove all the detectable pulmonary lesions. Additional indications for lung metastasectomy are the existence of effective systemic chemotherapy as a combined modality, difficulty of differential diagnosis from primary lung cancer, no other effective treatment except for resection or symptomatic pulmonary metastases, e.g., pneumothorax and haemoptysis [3]. Concerning the outcome, several factors are associated with prolonged disease-free survival and overall survival, such as primary tumour histopathology; the number, size and site of metastases; as well as a long progression-free interval between successful treatment of the primary cancer and pulmonary metastasectomy [4].

Another important aspect to consider when performing a lung metastasectomy consists of the surgical technique and approach. Indeed, in case of multiple lesions, it is important to adopt the most lung-sparing approach possible to preserve pulmonary function (and, consequently, the quality of life) and to spare the lung from potential additional lung resections.

The traditional pulmonary wedge resection can be performed in an open or thoracoscopic approach, and it can be carried out by a mechanical stapler. By the way, mechanical stapling, because of the proper morphology of the stapler, makes it impossible to spare as much lung parenchyma as needed above all for centrally located lesions. Indeed, in these last cases, some surgeons may prefer to perform a lobectomy rather than a wedge resection to achieve free surgical margins.

It is also possible to perform resection with diathermy coagulation; however, this technique does not provide a reliable haemostatic and air-sealant effect [5], especially for large and centrally located lesions.

In this framework, laser technology has been introduced in recent decades in several parts of Europe and other countries. Thanks to the capability of the laser beam to accurately cut the lung, laser-assisted metastasectomy may potentially overcome the technical limits of mechanical stapling of lung resections. Some authors reported satisfactory results with laser-assisted resection in terms of feasibility, low-rate peri-operative complications, completeness of resection and good oncologic outcomes [6].

Some issues are of main concern when using laser to perform lung resections, for example, airtightness after non-anatomical parenchymal lung resection with a Nd: YAG laser [7]. Analysing the current data, it is possible to conclude that laser resection is useful, and its role is important in case of multiple disseminated disease or deep-seated lesions. However, the majority of treatments using laser-assisted metastasectomy have been reported with a (mini)thoracotomy approach, while only few centres have combined laser-assisted PM with a mini-invasive approach (VATS or RATS). In particular, there are very limited data about the use of laser resection with uniportal VATS. The main focus of this manuscript is to report our institutional experience in performing lung-sparing laser-assisted PM by U-VATS, which we labelled as uniportal laser-assisted VATS (U-LA-VATS). The surgical technique and peri-operative results from our series of patients were herein presented and compared with the pertinent literature.

## 2. Materials and Methods

### 2.1. Patients Characteristics and Indication to Surgery

Between March 2021 and November 2023, among 98 patients who underwent non-anatomical PM, a total of 22 patients (18 men (75%); 6 women (25%); mean age 61.4 years; age range 13–83 years) were treated with laser-assisted PM at our institution. Patients who underwent anatomical resection were excluded for the purpose of the analysis (see Supplementary Table S1). Two patients received surgery twice; for this reason, we had a total of 100 resections, of which 24 were laser-assisted (CONSORT diagram, Supplementary Table S1).

Lung nodules were suspected on the basis of radiological characteristics or lab tests (i.e., CEA levels), and a confirmatory biopsy was performed in case of doubt or in the case of patients who may benefit from neoadjuvant treatment. All cases were discussed in a multidisciplinary tumour board, and the indication to surgery was proposed in agreement with oncologists and radiation oncologists. The majority of patients had a history of neoplasm with colic adenocarcinoma as the most common tumour. Two patients had no previous neoplastic history, and, in their cases, the choice of laser resection was driven by the position of the nodule (deep and central), with a pre-operative poor pulmonary function.

The standard surgical approach consisted of a mini-invasive thoracoscopic approach (UVATS #56 cases; see details below) when the lesions were peripherally located in the lung parenchyma. In case of deeply located or multiple small (<5 mm) lesions, a (mini)thoracotomy (#42 cases) was performed at the 5th intercostal space.

Laser-assisted PM (see details below) was indicated according to the surgeon’s choice and only when the lesion was far from great vessels or pulmonary artery/vein or bronchus. A combined approach (laser-PM via UVATS) was proposed only in selected cases according to the number and the lesion location.

Prolonged air leak was defined in case of the presence of air leak after the 3rd post-op day after surgery. All patients underwent electrocardiography, routine lab tests and functional pulmonary tests before surgery to better stratify the post-operative risk.

### 2.2. Statistics

Descriptive statistics were calculated and expressed as mean ranges and standard deviation. All the clinic pathological variables are summarized in Table 1 and analysed in a univariate analysis (Table 2). For categorical variables, the Fisher association test was used, and for continuous variables, the T test was used. The analysis was performed using SPSS 26 (IBM SPSS Statistics, IBM Corporation, Chicago, IL, USA).

### 2.3. Surgical Technique

All surgeries are performed in general anaesthesia and single-lung ventilation. The patient was placed on lateral decubitus, kept in position by a vacuum mattress and with both arms flexed and stretched toward their head [8]. The intercostal spaces can be widened by putting a roller blanket under the patient, at the nipple level, and by flexing the surgical table in a wedge-shape position [8]. Usually, a 3–4 cm single incision, totally muscle-sparing, is performed at the V intercostal space on the middle axillary line. A wound protector (Alexis Wound Retractor, Applied Medical, CA, USA) is used to prevent the spread of neoplastic cells in subcutaneous tissues during the removal of the lung specimen and to keep the camera clean during its introduction through the incision [8,9]. A rib spreader or additional accesses are not required.

The 30° 10 mm camera is introduced in the upper part of the incision (that represents the fulcrum of the camera shank during its movements), while all the other instruments are introduced below it. To improve the space for the instruments under the camera area, the operating table can be tilted towards the ventral side of the patient (where the surgeon stands) by 20–30 degrees [9] and gently in an anti-Trendelenburg position.

In Uniportal VATS, dedicated instruments are used, characterized by a long shape and a curved shape, with distal and proximal articulations. These characteristics allow for their simultaneous introduction through the incision and use without the risk of fencing if some basic rules are respected. In particular, the lung graspers are introduced in the middle part of the incision, just below the camera, while the main instrument used in that moment, as the thoracoscopic laser probe, must be introduced in the lowest part of the incision [9]. 

Uniportal VATS allows the surgeon a quite easy digital palpation of the lung nodules through the single incision [8]. This aspect gives the possibility for the surgeon to better localize the nodule to be ablated by the laser probe and to intraoperatively evaluate the macroscopic-free margins around the nodule. A single 28Fr chest tube is left in place at the end of the operation, introduced through the same incision [8].

A surgical laser system (Opera EVO, Thulium + Diodo OUTPUT 30–10 W, Quanta System S.p.a., Samarate (VA), Italy), with a wavelength of 2010 nm, 150 W maximum power and a touch-screen display, was used. The energy was delivered in a continuous mode by a 550 μm sterile optical fibre conducted through a specific handpiece (25 cm in length, malleable, 16 G and 1.1 mm in diameter) and activated by a footswitch. The handpiece is provided in combination with a high-performance rigid endosuction that is extremely useful to eliminate vaporization fumes.

After laser-assisted lung resection, if subsequent underwater test for air leak was negative, no suture is performed at the level of the surgical site. Otherwise, an over-suture with the V-lock 3-0 stitch is performed for aerostatic purpose. 

As for VATS procedures, even in U-LA-VATS procedures, a single 28Fr chest tube is left in place at the end of the operation, introduced through the same incision.

## 3. Results

In 12 cases (50%), thulium laser-assisted resection was performed using uniportal video-assisted thoracic surgery (U-LA-VATS), and in the remaining cases (12, 50%), laser-assisted PM was performed by a thoracotomic approach (lateral muscle-sparing thoracotomy and mini-thoracotomy in equal parts) (see Table 1). All the resections were not anatomical (atypical o wedge resections). Out of the 22 patients, 4 were operated twice (one for each side), and all of these cases previously had colic adenocarcinoma.

The general mean duration of surgical intervention was 110.5 ± 54.1 min. In the thoracotomy group, the mean duration of surgery was 95 ± 57.7 min, and it was 73.5 ± 35.5 min in the uniportal VATS group. At the univariate analysis, this difference resulted to be statistically significant (*p* value 0.025). According to the indication to the different approach, it was not surprising that the mean duration of VATS resection was shorter because one of the main indications to open resection was the presence of multiple nodules. 

Chest tube stay was shorter in the uniportal VATS group (2.9 ± 0.3 than in the thoracotomy group (3.5 ± 0.6), but this difference was found to be not statistically significant (*p* = 0.084). Mean hospitalization after surgery was 2.9 ± 0.3 days for the uniportal VATS group and 3.7 ± 0.9 days for the thoracotomy group; this difference was statistically significant according to the results of the univariate analysis (*p* value 0.015). 

No major post-operative complications were observed; in particular, prolonged air leak (>3 days) was observed in only one laser-assisted lung resection (the thoracotomy group), where the chest tube was safely removed on the 5th post-op day.

All the removed lesions were confirmed to be lung metastases. In particular, they originated from colon/rectum cancer (19, 79.2%), soft tissue sarcoma (3, 12.5%) and cervix cancer (2, 8.3%). In the four cases bilaterally operated, both sides had metastases from colon adenocarcinoma. Metastases were single in 9 cases (75%) and multiple in 3 cases (25%) in the uniportal VATS group, and they were single in 4 cases (33%) and multiple in 8 cases (67%) in the thoracotomy group. Mean tumour dimension was 1.6 ± 0.4 cm in the uniportal VATS group and 1.1 ± 0.8 cm in the thoracotomy group.

Surgical margins were 0.5 ± 0.1 cm and 0.7 ± 0.5 cm for the uniportal VATS and thoracotomy groups, respectively.

## 4. Literature Review

### 4.1. Search Strategy and Eligibility Criteria

With the aim of comparing our experience with the data existing about this topic, a literature review was performed.

**Figure 1:**
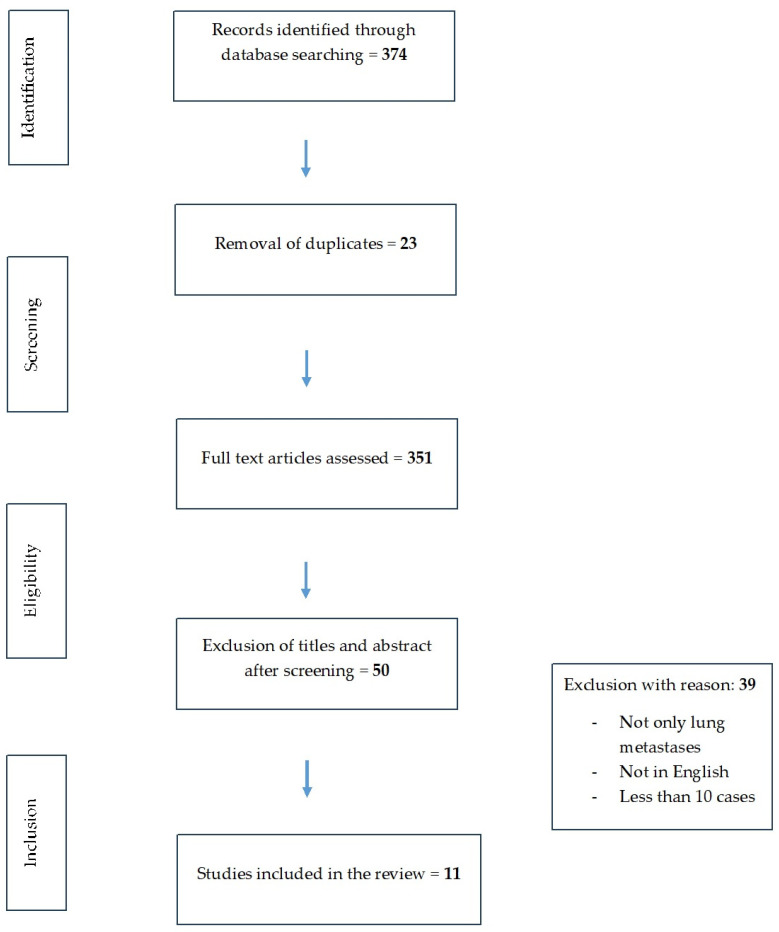
Preferred Reporting Items for Systematic Reviews and Meta-Analysis flow diagram for literature search.

Criteria that were used to lead the review are reported in line with the Preferred Reporting Items for Systematic Reviews and Meta-Analyses (PRISMA) statement (Figure 1). 

Comprehensive research of the literature was conducted on PubMed from 2000 to 2024. The advanced tool for the title and the abstract was used with the following keywords: laser, resection, lung. 

Year of publication: Any publication date starting from 1 January 2000 to 31 January 2024 was eligible. Language: Only studies with full text in English language were included.

Type of study: Only peer-reviewed publications reporting primary data were eligible. Therefore, reviews, editorials, letters and other forms of secondary expert opinion were excluded during the screening stage. Only full manuscripts were eligible, excluding conference abstracts and proceedings. No constraints were imposed based on the level of evidence.

We included all original studies describing the use of laser for lung resection on lung metastases in humans and reporting at least one post-operative outcome among the following: the number of total resections, the number of laser resections, the mean number of removed nodules, the rate of recurrence, the mean operation time, the mean chest tube duration and major complications.

The following data were extracted onto a Microsoft Excel 2022 spreadsheet: author, period, country, kind of study, number of patients, number of total resections, number of laser resections, kind of laser used, presence of the control group, mean number of removed nodules, approach, development of local recurrence, mean operation time, chest tube stay, development of major complication and bilateral surgery. The final manuscript was shared with different principal investigators of eligible studies (co-authors of the present study), and the final manuscript was approved by all co-authors. 

Exclusion criteria included the following: use of laser for other indications than lung metastasectomy (i.e., primary lung cancer, lung volume reduction surgery), endoscopic or percutaneous use of laser, case reports, case series and review.

### 4.2. Literature Research Outcome 

During the comprehensive literature search on PubMed, 374 articles were found. By reviewing titles and abstracts, articles formatted as reviews, editorials, letters, commentaries or case reports, 23 duplicates and non-English language articles were excluded. Forty-two eligible studies were selected and retrieved in full-text version; no additional study was found by cross-reference.

Thirty-nine full-text reports were excluded for the following reasons: reports included not only lung metastases, were not in English and involved fewer than 10 cases. Finally, 11 studies met all the inclusion criteria and were selected for meta-analysis [10,11,12,13,14,15,16,17,18,19,20] (Figure 1).

### 4.3. Characteristics of Selected Studies and Methodological Quality

Table 3 shows the general characteristics of the included studies. All the studies analysed at least 10 cases, except for one, from Loughlin and colleagues [10,19], who retrospectively described their first seven cases in their initial experience with laser resection. Out of the other 10, 5 [10,13,14,16,17] described only laser resection cases, 3 described retrospective cases [10,13,16,17] and 1 described a prospective case [14]. The remaining 5 [11,12,15,18,20] were all retrospective studies, comparing laser with at least one other resection technique. Overall, 1260 laser resections were considered. Six studies were from Germany, two from Italy, one from China, one from Japan and one from Ireland. 

The methodological quality of the included papers was evaluated with the MINORS tool (Table 4).

### 4.4. Laser Type and Indication to Surgery

Table 5 shows the operatory characteristic of the selected studies. The most commonly used type of laser is the neodymium-doped yttrium aluminium garnet (Nd:YAG) laser, which was used in 9 out 11 cases. Of the 2 other cases, one used thulium laser, and the other one used potassium-titanyl-phosphate (KTP). The choice of laser resection was motivated by the presence of multiple nodules with deep location for Franzke [11]. Stefani [18] chose laser to spare parenchyma in deeply located nodules as an alternative to lobectomy or in case of multiple disseminated nodules.

Concerning the practical use of laser, Hassan and colleagues [14] defined a mean distance of 30 mm of resection and >30 mm for coagulation. Stefani et al. [18] used laser beam in a noncontact mode, with power output between 40 and 100 W. When the beam was very close to lesion, laser power was reduced to 40 W. The maximum focus of the beam was achieved at a distance of 30 mm. Loughlin and colleagues [19] described the power use of 40 W, often increased to 50 W. Nagayasu et al. [16], who used KTP laser, applied the laser beam on the parenchymal bed at the resection margin to achieve coagulation. Liu and colleagues [12], the only ones who used thulium laser, applied no contact mode with 40 W power for resection and 20 W power for coagulation on the bed of resection. The other 6 studies did not report details on the power used.

With oncological radicality given as the main purpose for surgery, Nagayasu [16] took into account 2.5 cm and Liu [12] 2 cm. Porrello [17] considered 5–10 mm as a safe margin. For Stefani [18], a 4–5 mm margin was considered. Rolle et al. [10] took in consideration 3 mm for the resection plus 5 mm of necrosis of the resection bed. The other 4 studies did not provide details on safety margins, but Franzke and coworkers pointed out that they obtained 100% margins with laser resection.

### 4.5. Surgical Approach for Lung Metastasectomies

Concerning the surgical access to the pleural cavity, the surgical access was in open technique, with anteroaxillary in two cases and thoracotomy in 6 cases. In two of the selected articles, Liu and colleagues and Loughlin and colleagues [12,20] associated the use of laser for lung resection and the minimally invasive technique bi-VATS in both cases.

Meyer [13] performed an anterior minithoracotomy incision (approximately 5–7 cm length) at the fifth intercostal space through which they applied a soft-tissue retractor (Alexis Protector; Applied Medical). Two additional working ports were inserted. The entire lung was palpated via minithoracotomy. All detected lung metastases were removed under thoracoscopic control. In the series from Schmid et al. [15], laser resection was performed via thoracotomy.

Schimd, Moneke, Stefani, Nagayasu and coworkers [15,19,20] described their series in which they performed wedge resection in all cases, all using the thoracotomy approach. In Franzke and Hassan [11,14] series, all resections were carried out via an open approach through anterolateral thoracotomy or minimally invasive video-assisted thoracoscopic surgery when feasible. Rolle, Porrello and colleagues [10,17] used an anteroaxillary muscle-sparing approach in all cases. The mean operation time was reported in only 4 studies [12,13,14,18] and was 118.1 ± 42.6, 102, 129 and 114 min, respectively.

The number of removed nodules per patient has been quite variable, with an average going from 1 to 8 nodules per patient.

Chest tube stay was variable, always shorter than 7 days, with the shorter stay for patients who were operated with bi-VATS access (1 and 2 days in the two series that described the bi-VATS access). The most frequent post-operative complication was air leak.

## 5. Discussion

The use of laser for lung nodules is known to be a useful approach for deeply located or multiple nodules. Different kinds of laser are available, such as Nd:YAG laser or thulium laser, with the Nd:YAG laser being the most used according to literature records we went through in the literature search made for the reported review. Laser can be used either in open or minimally invasive technique. In particular, data present in the literature report the spreading use of the minimally invasive approach. All the reported experiences, by the way, describe a multiportal approach.

Careful study of the pertinent literature was of utmost importance to fully understand the key point of our approach compared with the presented works. Indeed, without a deep analysis of the selected paper, we would have not been able to underline that probably the strongest point of our series is that all VATS approaches are performed through a single incision.

In our series, nearly half the patients have been operated with the uniportal video-assisted thoracoscopy technique. This let us draw the first important conclusion: our series is the only one, according to the best knowledge available in the literature, in which laser-assisted resection has been performed, when minimally invasive, through a single port.

Together with the series described by Liu [12], ours is the only one in which thulium laser was used. Concerning the indication for this kind of surgery, usually it has been used for metastases that were, in the majority of cases, from colon adenocarcinoma, and this aspect was present in our series too. Metastases were usually multiples in the thoracotomy group; indeed, this kind of technique makes it easier to find and remove multiple nodules with safe margins, thanks to the direct palpation of the lung parenchyma. In our series, we can confirm this kind of indication; indeed, the number of nodules was nearly 2 for U-VATS resection and nearly 4 for the open approach, confirming that the resection of multiple metastases is more feasible through direct lung palpation. According to the indication to a different approach, it was not surprising that the mean duration of VATS resection was shorter if compared with the open approach because the mean number of removed nodules is smaller in the VATS approach. Furthermore, the length of the incision to close for the uniportal VATS is, of course, shorter than that for thoracotomy, and this contributes to a shorter surgical time for the uniportal VATS approach. In our series, the difference of the mean duration of surgery was statistically significant for UVATS vs. open surgery.

The principal complication described in the literature is air leak, which can be overwhelmed by applying a continued suture over the resected parenchyma or sealant materials. In our series, no air leak after surgery was detected after uniportal laser-assisted resection, while only 1 case (8.3%) was detected after in the thoracotomy group. This aspect is very important for this kind of surgery. Indeed, air leak is usually one of the most common complications after lung resection. This risk may increase after multiple lung resections. Furthermore, it should not be forgotten that these are usually metastatic patients and that, after surgery, they may go to adjuvant therapy. The eventual development of air leak may cause a delay in post-operative oncological treatment. For this reason, we considered this particular outcome of utmost importance in a comprehensive view of the oncological treatment. In our series, no air leak was detected after surgery.

Chest tubes were usually removed on the 2nd or 3rd post-operative day, with an advantage under this point of view for U-VATS resection rather than open access. Surgical margins were free from disease in all cases, so oncological radicality was kept. In our series, we had a relapse of disease on the margin of the previous resection in only 1 case, and it was in the case performed via thoracotomy.

The other consequence of shorter surgery time and short chest tube stay is shorter hospitalization time. In particular, for UVATS surgery in our cohort, the mean hospital stay was 3 days vs. a mean of 4 days for open surgery. This difference was statistically significant.

Our series describes the use of lung resection for metastatic nodules both in open and VATS approaches, being the first to perform minimally invasive laser resection in U-VATS. Peri-operative and oncological outcomes were compared with the literature records, confirming safety and feasibility of this approach. We are absolutely aware that our results are affected by some limitations: the monocentric and retrospective nature of this study and the small number of patients (only 12 in the cohort of the U-VATS laser resection). Validation studies with a greater number of patients are mandatory to eventually confirm these findings to implement the use of laser for lung resection for well-selected cases, with the chance to perform this kind of lung-sparing resection through a single port.

## 6. Conclusions

Uniportal laser-assisted video-assisted thoracoscopy (U-LA-VATS) was demonstrated to be a safe and effective procedure above all for multiple nodules or deeply located ones, with a shorter operation time and shorter hospital stay after surgery, when compared with laser resection via an open approach. Compared with the selected works for the review, our series is the only one describing the use of laser resection with this kind of minimally invasive approach. Further prospective studies with a greater number of cases are desirable to validate our preliminary findings to implement the use of laser resection.

## Figures and Tables

**Table 1:** Surgical features of our patients.

	Thoracotomy (*n* = 12–50%)	VATS Uniportal (*n* = 12–50%)
Sex		
M	11 (92%)	7 (58%)
F	1 (8%)	5 (42%)
Age at surgery (Mean ± sd)	56.7 ± 16.1	67.1 ± 12.2
Intervention duration (min) (Mean ± sd)	95.0 ± 57.7	73.5 ± 35.5
Drainage duration (days) (Mean ± sd)	3.5 ± 0.6	2.9 ± 0.3
Major Complications		
NO	12 (100%)	12 (100%)
YES	0 (0%)	0 (0%)
Minor Complications		
NO	9 (75%)	11 (91.7%)
YES	3 (25%)	1 (8.3%)
Air leakage post-surgery		
NO	11 (91.7%)	10 (100%)
YES	1 (8.3%)	0 (0%)
Hospitalization days after surgery (Mean ± sd)	3.8 ± 0.9	2.9 ± 0.3
Origin of lung metastases		
Lower gastrointestinal tumour	11 (92%)	8 (66.7%)
Soft tissue sarcoma	1 (8%)	2 (16.7%)
Cervix cancer	0 (0%)	2 (16.7%)
Metastasis		
Single	4 (33%)	9 (75%)
Multiple	8 (67%)	3 (25%)
Tumour location		
Left upper lobe	3 (25%)	4 (33.3%)
Left lower lobe	2 (16.7%)	1 (8.3%)
Right upper lobe	2 (16.7%)	4 (33.3%)
Middle lobe	1 (8.3%)	2 (16.7%)
Right lower lobe	4 (33.3%)	1 (8.3%)
Major lesion size (cm) (Mean ± sd)	1.1 ± 0.8	1.6 ± 0.4
Surgical margins (cm) (Mean ± sd)	0.7 ± 0.5	0.5 ± 0.1
Relapse on “surgical bed”		
NO	12 (100%)	11 (92.7%)
YES	0 (0%)	1 (8.3%)
Smoker		
NO	1 (8.3%)	6 (50%)
YES	3 (12%)	2 (16.7%)
Former	8 (66.7%)	4 (33.3%)
COPD		
NO	10 (83%)	11 (92.7%)
YES	2 (17%)	1 (8.3%)
Diabetes		
NO	10 (83%)	10 (83.3%)
YES	2 (17%)	2 (16.7%)
Cardiovascular disease		
NO	10 (83%)	6 (50%)
YES	2 (17%)	6 (50%)

**Table 2:** Univariate analysis.

	U-VATS (%)	Open Approach (%)	*p*-Value
**Age at surgery (Mean** **± SD)**	67.1 (12.2)	56.8 (16.1)	0.111
**Sex**			0.135
**F**	5 (41.7)	1 (8.3)	
**M**	7 (58.3)	11 (91.7)	
**Intervention duration (min) (Mean ± SD)**	73.5 (35.5)	95.0 (57.7)	0.025 *
**Hospitalization days after surgery (days) (Mean ± SD)**	2.9 (0.3)	3.8 (1.0)	0.015 *
**Drainage duration (days)**			0.084
**2**	2 (16.7)	0 (0.0)	
**3**	10 (83.3)	7 (58.3)	
**4**	0 (0.0)	4 (33.3)	
**5**	0 (0.0)	1 (8.3)	
**Smoke**			0.115
**NO**	6 (50%)	1 (8.3)	
**YES**	2 (16.7%)	3 (25.0)	
**Former**	4 (33.3%)	8 (66.7)	
**Cardiovascular disease**			0.074
**NO**	6 (50%)	10 (83.3)	
**YES**	6 (50%)	2 (16.7)	
**COPD**			0.481
**NO**	11 (92.7%)	10 (83.3)	
**YES**	1 (8.3%)	2 (16.7)	
**Diabetes**			1
**NO**	10 (83.3%)	10 (83.3)	
**YES**	2 (16.7%)	2 (16.7)	
**Number of metastases** **(Mean ± SD)**	1.9 (2.3)	3.7 (2.7)	0.133
**Dimension (cm)** **(Mean ± SD)**	1.7 (0.4)	1.1 (0.8)	0.116
**Surgical margins (cm)** **(Mean ± SD)**	0.5 (0.1)	0.7 (0.5)	0.36

* *p*-Value statistically significant.

**Table 3:** Characteristics of included studies.

Author	Period	Country	Kind of Study	N Patients	N Total Resection	N Laser Resection	Kind of Laser	Control Group (n)
**Rolle** [10]	1996–2003	Germany	Retrospective cohort	328	3267	3267	Nd: YAG	No
**Franzke** [11]	2010–2015	Germany	Retrospective	178	236	115	Nd: YAG	Traditional lung resection (79)
**Liu** [12]	2015–2018	China	Retrospective cohort	120	120	60	Thulium	Traditional lung resection (60)
**Meyer** [13]	2016	Germany	Retrospective	15	29	29	Nd: YAG	No
**Hassan** [14]	2017–2019	Germany	Prospective	61	77	77	Nd: YAG	No
**Schmd** [15]	2005–2016	Germany	Retrospective cohort	106	106	46	Nd: YAG	Traditional lung resection (60)
**Nagayasu** [16]	1998–2002	Japan	Retrospective	26	26	26	Potassium-titanyl-phosphate (KTP)	No
**Porrello** [17]	1995–2009	Italy	Retrospective	209	106	106	Nd: YAG	No
**Stefani** [18]	2005–2017	Italy	Retrospective cohort	89	42	42	Nd: YAG	Traditional lobectomies (47)
**Loughlin** [19]	2/2017–10/2017	Ireland	Retrospective	7	8	8	Nd: YAG	No
**Moneke** [20]	2005–2016	Germany	Retrospective cohort	77	705	438	Nd: YAG	Traditional lung resection

**Table 4:** Assessment of methodological quality of included studies with the Methodological Index for Nonrandomized Studies (MINORS) score (0 not reported, 1 reported but inadequate, 2 reported and adequate).

Item	Rolle [10]	Franzke [11]	Liu [12]	Meyer [13]	Hassan [14]	Schmid [15]	Nagayasu [16]	Porrello [17]	Stefani [18]	Loughlin [19]	Moneke [20]
**A clearly stated aim**	1	2	2	2	2	2	2	2	2	2	2
**Inclusion of consecutive patients**	2	2	2	0	0	2	2	2	2	2	2
**Prospective collection of data**	1	1	0	0	2	2	0	1	1	0	1
**Endpoints appropriate to the aim of the study**	2	2	2	2	2	2	2	1	2	2	2
**Unbiased assessment of study endpoint**	0	0	0	0	0	0	0	0	0	1	1
**Follow-up period appropriate to the aim of the study**	0	0	2	2	2	0	2	2	1	0	2
**Loss to follow-up less than 5%**	0	0	1	2	1	0	2	2	0	0	0
**Prospective calculation of the study size**	0	0	0	0	0	0	0	0	0	0	0
**Additional items in the case of comparative study**											
**An adequate control group**		2	2			2	2		2		2
**Contemporary groups**		2	2			2	2		1		2
**Baseline equivalence of groups**		1	1			1	1		1		1
**Adequate statistical analyses**		1	2			1	1		2		2

**Table 5:** Surgical Features of Selected Studies’ Patients.

Author	Mean of Removed Nodules	Approach	Local Recurrence	Mean Operation Time	Chest Tube Stay (Days)	Major Complication
**Rolle** [10]	8	Anteroaxillary thoracotomy			4.79	Prolonged air leak, bleeding,
**Franzke** [11]	3.28	Anterolateral thoracotomy	0.8%			
**Liu** [12]		bi-VATS	0	118.1 ± 42.6	2.0 ± 2.4	Air leak, bleeding
**Meyer** [13]	2	Minithoracotomy with two additional ports	0	102		
**Hassan** [14]	2	Thoracotomy, VATS		129 (55–334)	4	Air leak
**Schmid** [15]	6.5	Thoracotomy	0,14			Air leak, bleeding
**Nagayasu** [16]		Thoracotomy			1.8 ± 1 and 3.6 ± 2.9	Air leak
**Porrello** [17]		Anteroaxillary thoracotomy	0			Air leak
**Stefani** [18]	1	Thoracotomy	3	114	2.2	Air leak (only patients in which they did not suture the bed of the resection)
**Loughlin** [19]	1	bi-VATS			1	Air leak
**Moneke** [20]		Anterolateral thoracotomy	3		<7	Pneumonia, atrial fibrillation

## Data Availability

Data are available from the authors upon request.

## Supplementary Materials

The following supporting information can be downloaded at: https://www.mdpi.com/article/10.3390/jcm13185346/s1, Table S1: Patients selection CONSORT diagram.
