# Supplementary material for: Uniportal Laser-Assisted Video-Assisted Thoracoscopy (U-LA-VATS) for Lung Metastasectomy: Technical Description, Peri-Operative Results and Pertinent Literature Review
Source: J Clin Med. 2024 Sep 10;13(18):5346. doi: 10.3390/jcm13185346 (PMC11432019; doi:10.3390/jcm13185346)
Supplement: Supplementary file 1 [file jcm-13-05346-s001.zip › jcm-3143913-supplementary.pdf]

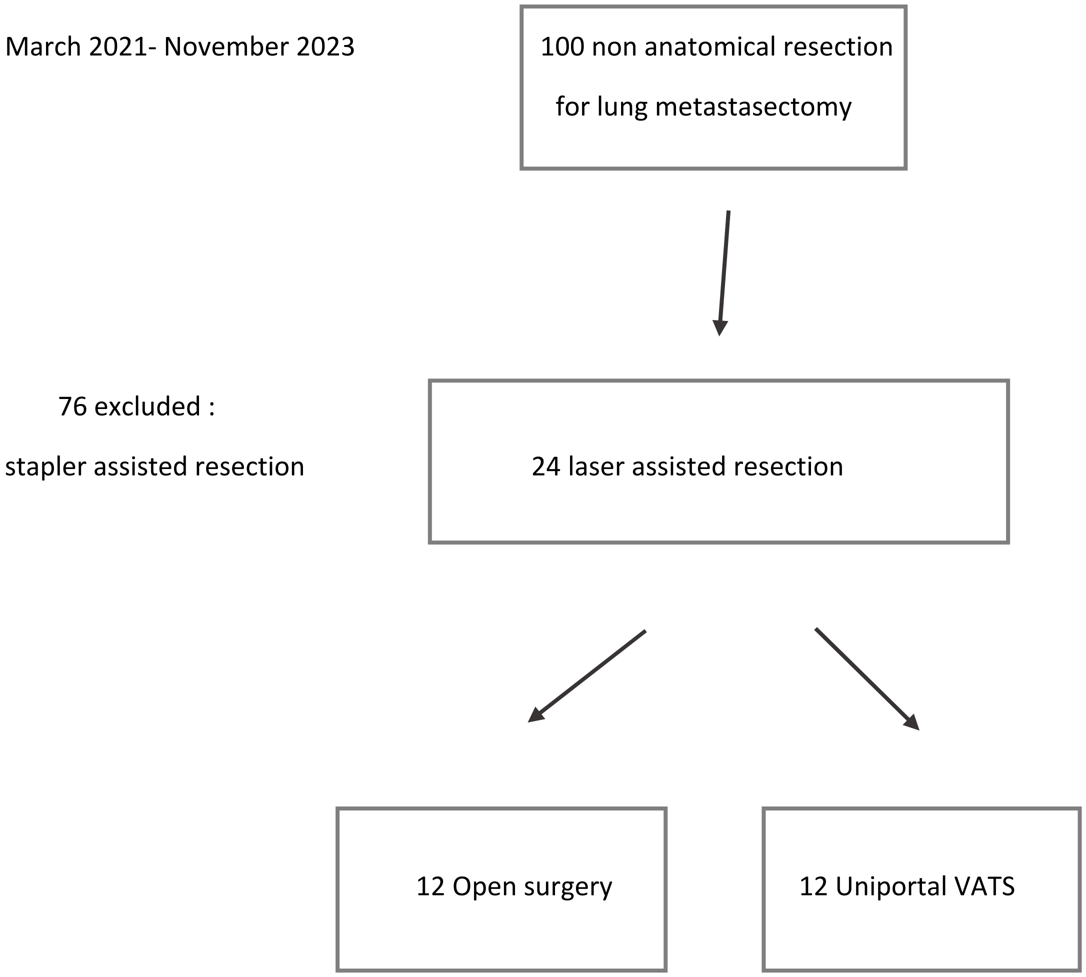

**Origin of lung metastases**		
Lower gastrointestinal tumor	11	8
Soft tissue sarcoma	1	2
Cervix cancer	0	2

Supplementary Table S1. Patients selection CONSORT diagram
